# Cytotoxicity on Human Cancer Cells of Ophidiacerebrosides Isolated from the African Starfish *Narcissia canariensis*

**DOI:** 10.3390/md8122988

**Published:** 2010-12-22

**Authors:** Fereshteh Farokhi, Gaetane Wielgosz-Collin, Monique Clement, Jean-Michel Kornprobst, Gilles Barnathan

**Affiliations:** 1 Research Group Sea, Molecules and Health EA 2160, College of Pharmacy, University of Nantes, 1 rue Gaston Veil, BP 53508, F-44035 Nantes Cedex 01, France; E-Mails: fereshteh.farokhi@etu.univ-nantes.fr (F.F.); wielgosz-collin@univ-nantes.fr (G.W.-C.); kornprobst.jean-michel@neuf.fr (J.-M.K.); 2 INSERM CRNA U892, 8 quai Moncousu, BP 70721, F-44007 Nantes Cedex, France; E-Mail: monique.clement@univ-nantes.fr (M.C.)

**Keywords:** glycosylceramides, human cancer cell lines, *in vitro* anticancerous activity, *Narcissia canariensis*, starfish

## Abstract

The starfish *Narcissia canariensis* harvested from the coasts off Dakar, Senegal, was investigated for glycolipids (GL). This report deals with the isolation, characterization and biological activity of a fraction F13-3 separated from the GL mixture and selected according to its ability to inhibit KB cell proliferation after 72 hours of treatment. Firstly, a GL mixture F13 was obtained that accounted for 1.36% of starfish biomass (dry weight) and 0.36% of total lipids. The fraction F13-3 obtained from F13 contained three homologous GL identified as peracetylated derivatives on the basis of chemical and spectroscopic evidence. These contained a β-glucopyranoside as sugar head, a 9-methyl-branched 4,8,10-triunsaturated long-chain aminoalcohol as sphingoid base and amide-linked 2-hydroxy fatty acid chains. The majority (63%) had an amide-linked 2-hydroxydocosanoic acid chain and was identified as the ophidiacerebroside-C, firstly isolated from the starfish *Ophidiaster ophidiamus*. The minor components of F13-3 differed by one more or one less methylene group, and corresponded to ophidiacerebroside-B and -D. We found that F13-3 displayed an interesting cytotoxic activity over 24 hours on various adherent human cancerous cell lines (multiple myeloma, colorectal adenocarcinoma and glioblastoma multiforme) with an IC_50_ of around 20 μM.

## 1. Introduction

Nowadays, myeloma, glioblastoma and carcinomas are a real public health problem in the world with increasing mortality rates in developing countries.

Multiple myeloma (MM) is a cancer of the white blood cells known as plasma cells; it is characterized by skeletal destruction, renal failure, anemia and hypercalcemia. Despite progress in the management of patients, MM remains an incurable disease, with a five-year survival rate not exceeding 50%. Recent advances in the understanding of the pathobiology of multiple myeloma (MM) have provided the basis for a more comprehensive effort to develop novel therapies for this disease [1,2]. As myeloma cells develop mechanisms of resistance to most known treatments [3], the search for new efficient anti-cancerous compounds is needed. Glioblastoma is the most common and most aggressive type of primary brain tumor in humans, involving glial cells.

Glioblastoma multiforme (GBM) is the highest grade glioma (grade 4) tumor and the most malignant form of astrocytomas. In most European and North American countries, incidence is approximately 2–3 new cases per 100,000 people per year. This malignancy of the central nervous system is fatal despite treatment with surgery and adjuvant therapy. In the United States, GBM occurs at a frequency of approximately 5000 cases annually, and constitutes up to 80% of all malignant gliomas. Long-term control of these tumors is rarely achieved, despite surgical resection and external beam radiation therapy, and GBM recurs within 6–10 months with a median survival of approximately 12 months [4].

Carcinomas are invasive malignant tumors consisting of transformed cells arising from epithelial origin. Epithelial cells cover the external surface of the body, line the internal cavities and form the lining of glandular tissues. Carcinomas are classified by their histopathogical appearance referring to the putative cell of origin or primary organ. With more than 600,000 deaths worldwide per year, colorectal carcinoma is the fourth most common form of cancer in the United States while approximately 36,000 persons will be newly diagnosed with oral cancer in 2010 [5].

Glycosphingolipids (GSL) are ubiquitous membrane constituents in animals, which play a fundamental role in major phenomena such as cell-cell recognition and antigenic specificity [6,7]. In general GSL exhibit a wide range of biological functions that might be related to the amphipathic nature of the molecule. Several GSL and other various glycolipids (GL) have been isolated from a number of marine sources, mainly including sponges and echinoderms. Glycolipids are known to possess immunomodulating and antitumor activity in particular those isolated from sponges [6–9]. Among them, GSL represent a large group of biomolecules containing two basic structural units: a sugar linked to a ceramide. The hydrophobic ceramide portion involves a sphingoid base and an amide-linked fatty acyl chain. Various cerebrosides, glycosylceramides with a single sugar head, were isolated from sponges [6–9], tunicates [10], octocorals [11], and echinoderms [12–25]. The phylum Echinodermata comprises approximately 7000 living species and their cerebrosides in particular have been chemically studied but little is known regarding their biological and pharmacological properties.

In the search for new efficient GL against cancer, we investigated a not yet studied starfish, *Narcissia canariensis*, harvested off Dakar, Senegal. This paper reports on the isolation of a GL fraction containing particular GSL named ophidiacerebrosides and the evaluation of their cytotoxic activity against various human cancer cell lines.

## 2. Results and Discussion

### 2.1. Glycolipid Isolation and Structure Determination

**Figure 1:**
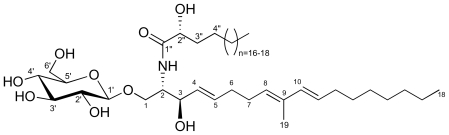
Glucosylceramides for *Narcissia canariensis*: ophidiacerebroside-B (*n* = 16), ophidiacerebroside-C (*n* = 17) and ophidiacerebroside-D (*n* = 18).

The common African starfish *Narcissia canariensis* was investigated for lipids and GL fractions. The lipid extract (8.97 g) obtained with CH_2_Cl_2_/MeOH was subjected to lipid class separation by column chromatography affording a crude GL fraction (0.92 g). A subsequent column chromatography enabled obtaining a bioactive GL fraction named F13 selected for its ability to inhibit KB cell proliferation significatively. The F13 fraction was in turn subjected to a further preparative chromatography that enabled obtaining a purified GL fraction named F13-3 as a white amorphous powder. It showed a single spot on thin layer chromatography with an Rf value similar to that of a commercial standard galactocerebroside with a hydroxylated acyl chain. Interestingly, fraction F13 represented 1.36% of the starfish biomass (dry weight), 0.36% of total lipids and 3.58% of the total GL. Fraction F13-3 contained a major GSL (63%) and two minor homologous ones as shown by NMR and mass spectrometry studies. Thus, the peracetylated F13-3 exhibited the characteristic signals of a sphingoid base and a β-glucopyranose in the ^1^H-NMR spectrum (Figure 1, Table 1). Its electrospray ionization mass spectrometry (ESI) showed three molecular ion peaks, corresponding to three glycosylceramides with three different α-hydroxylated fatty acyl chains. Indeed, the peracetylated major GSL component displayed an adduct ion [M + Na]^+^ at *m/z* 1084.6880 (high resolution ESI) in accordance with the formula C_59_H_99_NO_15_Na (a molecular mass of 809.7 amu for the intact GSL). The minor GSL of peracetylated F13-3 displayed sodiated molecular ions at *m/z* 1070.6702 and 1098.7051 in accordance with a methylene less or more than the major one. The structure of the major cerebroside was determined on the basis of chemical and spectroscopic evidence. Thus, this glycosylceramide contains a triunsaturated long-chain aminoalcohol as the principal sphingoid base. The optical rotation value of peracetylated F13-3 was determined as [α]_D_ ^30^ = +0.44 (*c* = 0.4, CH_2_Cl_2_).

All the chemical shifts of the ceramide are given in the ^13^C and ^1^H NMR spectra (Table 1). The sugar linked to the ceramide was identified as glucose by NMR spectroscopy. First of all, the anomeric proton of the β-glucopyranoside (δ = 4.49, d, *J* = 7.9 Hz) was correlated with the anomeric carbon at δ = 100.5 ppm in the HMQC spectrum. Starting from this proton, all the ^1^H and ^13^C NMR signals of the sugar were assigned by using the COSY, HMQC and HMBC spectra, and the vicinal proton-proton coupling constants were determined (Table 1). The *gluco* configuration of the sugar, as well as its β anomeric configuration, was established on the basis of the ring proton coupling constants (*J*_1,2_ = 7.9 Hz, *J*_2,3_ = 9.5 Hz, *J*_3,4_ and *J*_4,5_ = 9.6 Hz). The linkage of the glucopyranoside to the ceramide was confirmed by the three bond _13_C-^1^H couplings of anomeric C-1′ with H-1a and H-1b observed in the HMBC spectrum. The presence of signals for five olefinic protons and the singlet at δ = 1.73 ppm indicated that a methyl branch is linked to an olefinic carbon atom. The unsaturation pattern in the ^1^H NMR spectrum showed a multiplet at 5.34 ppm and a double doublet at 5.43 ppm, characteristic of a Δ^4^ sphingosine with *trans* configuration (*J* = 15.0 Hz), a doublet at 6.04 ppm (*J* = 15.4 Hz) for a *trans* Δ^10^ double bond, and multiplets at δ 5.59, 5.83 ppm. COSY correlations were observed between H-7 and H-8 and H-19 (Table 1). Further correlations were observed between olefinic signals at δ 5.83 (H-8) and 5.59 (H-11), and vicinal methylene groups at positions 7 and 12. And also we observed no correlation between the olefinic carbon C-9 and an olefinic proton in the HSQC spectrum. The key HMBC correlations from H_3_-19 to C-8, C-9, and C-10, and from C-11 to C-9, confirmed the location of the positions of the double bonds. These data allowed us to establish the olefinic pattern of the sphingoid unit as a 9-methyl-4,8,10-triene.

To determine the structure of the ceramide, F13-3 was subjected to an acidic methanolysis and the resulting reaction mixture was separated by partitioning between CH_2_Cl_2_ and H_2_O/MeOH into an aqueous phase containing methylglycosides and an organic phase containing 2-hydroxylated fatty acid methyl esters (FAME) and sphingoid bases. Thereafter the latter mixture was analyzed by GC/MS. Only one sphingoid base was observed.

The FAME mixture from the active fraction F13 was analyzed by GC-MS. The 2-hydroxy FAME produced the characteristic ions at *m/z* 90 (McLafferty) and *m/z* 103. The results were as follows: 2-OH-21:0, *t_R_* = 33.9 min (15.4%), *m/z* 356 (M^+^); 2-OH-22:0, *t_R_* = 36.4 min (63.6%), *m/z* 370 (M^+^); 2-OH-23:0, *t_R_* *=* 38.8 min (21.0%), *m/z* 384 (M^+^). These fatty acid structures were confirmed by GC-MS analysis of *N*-acyl pyrrolidides showing fragment ions at *m/z* 129 (McLafferty) and the expected molecular ions. The peracetylated methylglycoside from F13-3 was analyzed by GC-MS (column temperature 110 °C (2 min) and then (temp. increasing at 3 °C/min until 240 °C)); *t_R_* = 31.6 min (methylglucopyranoside) similar to that of an authentic sample. Other diagnostic ions were observed at *m/z* 331 (M-OMe)^+^, 303 (M-OAc)^+^, 243, 200, 157, 145 and 115.

These data showed that F13-3 contained a ceramide composed of the known 4,8,10-triunsaturated, 9-methyl branched C_18_ sphingoid base and 2-hydroxylated fatty acyl chains like cerebrosides isolated from other invertebrates [8,10,11] including starfish [16,19,20,22,25].

### 2.2. Cytotoxic Activity

The cytotoxic activity of F13-3, including ophidiacerebroside-C as major component, was detected and followed using KB cells (human oral epidermoid carcinoma) (IC_50_: around 20 μM after 72 h of treatment). Thereafter it was investigated on three human cancerous cell lines, KMS-11 (adherent plasma cells obtained from patients with multiple myeloma [26]), GBM (astrocytoma cells obtained after tumor resection of patients with glioblastoma multiforme-primary culture [27]), and HCT-116 (colorectal adenocarcinoma cells derived from a patient with Lynch’s syndrome [28] and as described in the experimental section). Results are shown in Table 2.

The activities observed, mainly on KMS-11 and HCT-116, are interesting as ophidiacerebrosides have not yet been evaluated on human cancer cells. Cytotoxicity on these three cell lines was already found in the same range of concentration for some synthetic bile acid derivatives (LD_50_: 8.5 μM) in a recent study [29]. A mixture of ophidiacerebrosides with C_20_ to C_24_ 2-hydroxyacyl chains, including the major ophidiacerebroside-C with an acyl chain C_22_ occurring at 40%, has been described to display strong cytotoxicity against L1210 murine leukemia cells *in vitro* [16]. Cerebrosides isolated from a tunicate, phalluside-1 and -2, contain the same triunsaturated sphingoid base and sugar head, but they differ in 2-hydroxyacyl chain lengths, C_16_ and C_18_ respectively. Interestingly, the latter cerebrosides were found inactive against human cancer cells including lung carcinoma (A 549), colon carcinoma (HT 29), and melanoma (MEL 28) [10]. In addition, cerebrosides named renierosides with the same sphingoid base and various monounsaturated 2-hydroxylated fatty acyl chains were found inactive against five human solid tumor cell lines [8]. These results suggest that the nature of the 2-hydroxylated fatty acyl chain (chain length and possible double bonds) seems to be important for the cytotoxic activities of this type of cerebrosides. Recently, it was shown that the nature of the sugar residue may be relevant for the biological activity of this type of GSL; those with glucopyranosides showing stronger cytotoxicity than those with galactocerebrosides [25]. Due to its potential biological interest, phalluside-1 found in the ascidian *Phallusia fumigata* [10], the sea stars *Allostichaster inaequalis* [20] and *Cosmasterias lurida* [19], has recently been recently synthesized [30].

In conclusion, this study provides an additional source (another starfish) for ophidiacerebrosides and points out the potential of these compounds against human cancerous cells. It would be of interest to investigate other GL fractions of *N. canariensis* for glycosylceramide isolation, in particular those with the same sphingoid base but differing by 2-hydroxylated acyl chain length and to compare their cytotoxic activities using the same panel of human cancer cell lines.

## 3. Experimental Section

### 3.1. General Procedures

High resolution electrospray ionization mass spectrometry (HR-ESI-MS, positive mode, ion-source acceleration 4.5 kV, ion-source temperature 200 °C, methanol as solvent) mass spectra were recorded with a Micromass Zab Spec Tof spectrometer. ^1^H- and ^13^C-NMR as well as 2D-NMR spectra were obtained on a NMR Bruker Avance-500 spectrometer with triple Probe TBI multinuclear in CDCl_3_ at 500.13 MHz and 125.76 MHz respectively, with reference to an internal standard of tetramethylsilane. Chemical shifts and coupling constants were expressed in δ (ppm) and Hz respectively. GC-MS spectra were performed on a Hewlett-Packard 6890 gas chromatograph with a mass selective detector MS HP 6890 MS, Little polar column DB-1, 60 m length × 0.25 mm i.d. × 0.25 μm phase thickness. The temperature of the column was varied, after a delay of 2–4 min from the injection, from 110 to 310 °C with a slope of 3 °C min^−1^. Optical rotations were measured in CH_2_Cl_2_ solutions with a Polartronic NH8 Schmidt/Haensch polarimeter at 30 °C. Analytical TLC was performed on precoated silica gel F_254_ plates. After development, the dried plates were sprayed with 50% H_2_SO_4_-vanillin and orcinol reagents.

### 3.2. Animal Material

**Figure 2:**
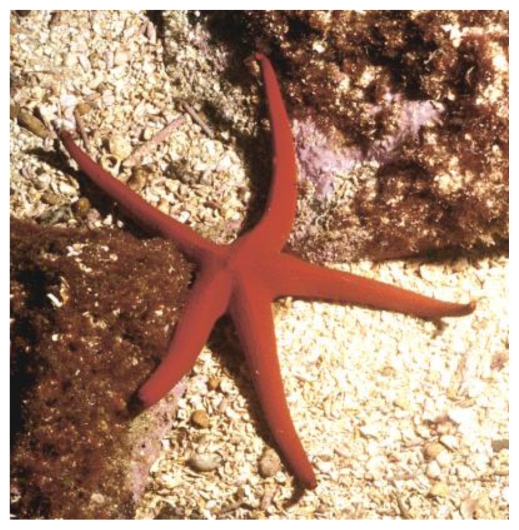
Narcissia canariensis.

The starfish *Narcissia canariensis*, shown below in Figure 2 (photo taken by Dr. Patrice Petit de Voize, Dakar) is found on rocks along the Senegalese coasts off Dakar, at a depth range of 23–38 m and were collected by hand during a scuba diving expedition organized by Oceanium of Dakar in 2009, on the sites named Petit Seminole, Fayss and Thi Wa. The specimens were identified by Professor Peter Wirtz, University of Madeira, Portugal.

### 3.3. Lipid Extraction and F13-3 Isolation

Whole bodies of the collected specimens (241.37 g dry weight) were chopped and twice extracted with CH_2_Cl_2_/MeOH (1:1, vol/vol) at room temperature. The combined extracts were concentrated *in vacuo* to give the crude extract, which was partitioned between H_2_O and CH_2_Cl_2_/MeOH. The organic layer was concentrated *in vacuo*, and the residue (8.97 g, 3.7%) was chromatographed on silica gel column with pure solvents as successive eluents: Dichloromethane (neutral lipids, 6.30 g), acetone (GL, 0.92 g) and methanol (phospholipids, 1.68 g). The GL mixture was separated on silica gel column to give 14 fractions. Among them, fraction 1 was subjected to a silica gel column chromatography (CH_2_Cl_2_/MeOH, 95:5 to 80:20, vol/vol) affording 23 fractions. From the latter fractions, fraction 13 (F13, 33 mg) gave a positive test on KB cells, and presented a similar polarity to a commercial standard (galactocerebroside with 2-hydroxy fatty acyl chain type I) (Rf = 0.35 on silica gel thin layer chromatography, CH_2_Cl_2_/MeOH, 88:12, vol/vol). Then F13 was subjected to silica gel chromatography with a solvent system of CH_2_Cl_2_ with 5% to 15% MeOH vol/vol) to give seven fractions. The following fraction 3 (21 mg), designated as F13-3, was obtained as a white amorphous powder and was used for biological studies. In order to determine the chemical structure, fraction F13-3 was peracetylated and studied by NMR and ESI-MS.

### 3.4. Acetylation of F13-3

A part of F13-3 (9 mg) was dissolved in 1 mL of acetic anhydride and some drops of dry pyridine. The reaction was allowed to proceed for 18 h in darkness at room temperature, and then the reaction mixture was partitioned between water and dichloromethane. The organic layer was washed with HCl 1 M, neutralized with a Na_2_CO_3_ solution, and dried on anhydrous sodium sulfate. The solvent was evaporated under reduced pressure and the residue was weighed.

### 3.5. Methanolysis of F13-3

A part of F13 (3 mg) was heated with 0.9 mL of MeOH/H_2_O/HCl (29:4:3, vol/vol/vol) at 80 °C for 18 h. The reaction mixture was extracted with H_2_O/CH_2_Cl_2_ (3:9, vol/vol), the aqueous layer was concentrated to give methylglycosides, whereas the organic layer contained a mixture of fatty acid methyl esters (FAME) and sphingoid bases. A part (1/3) of the FAME was preserved, the other one was transformed into *N*-acylpyrrolidides (NAP) by heating in a mixture pyrrolidine/acetic acid (10:2, vol/vol, 1 mL) during 1 h at 85 °C. The reaction mixture was separated with H_2_O/CH_2_Cl_2_ and the organic layer was dried on anhydrous Na_2_SO_4_, filtrated and weighed after solvent evaporation. The aqueous layer was neutralized by NaOH 1 M and extracted twice with diethyl ether. The organic layer, containing sphingoid base was dried and then acetylated. The aqueous layer containing methylglycosides was evaporated *in vacuo* and then acetylated before GC-MS analysis.

### 3.6. Cell Cultures

Cells were cultured in RPMI 1640 medium (KMS-11, GBM, HCT-116) or BME (KB) supplemented with 10% foetal calf serum, 2 mM glutamine, antibiotics (100 IU/mL penicillin and 100 μg/mL streptomycin) (Life Technologies). Cells were subcultured at confluency after dispersal with 0.025% trypsin in 0.02% EDTA. Cells were maintained in plastic culture plate at 37 °C in a humidified atmosphere containing 5% CO_2_. For experiments cells were used at 70–80% confluency.

### 3.7. Neutral Red Assay

**Figure 3:**
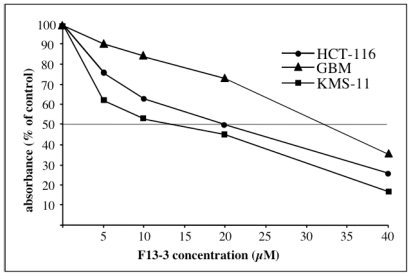
Dose-response curve of F13-3 (24 h of treatment).

For cytotoxicity tests, 20,000 cells (GBM, HCT-116) and 50,000 cells (KMS-11) (200 μL) were plated in 96-well culture microtiter plates (Falcon) and incubated at 37 °C in 5% CO_2_. After 24 h, drugs were added in 50 μL fresh medium, then after 21 h cells were loaded for 3 h with neutral red (3-amino-7-dimethylamino-2-methylphenazine hydrochloride) (Sigma-Aldrich, St Quentin, France) at a final concentration of 50 μg/mL in culture medium. Thereafter (24 h of treatment) the medium was removed, cells were fixed for 5 min with a mixture of 1% formaldehyde-1% CaCl_2_ and the dye extracted with 0.2 mL of 1% acetic acid in 50% ethanol. Plates were left overnight at 4 °C and absorbance was recorded at 570 nm (Multiskan EX-Thermo-Electron Corporation). Experiments were performed at least in triplicate, 4 wells per F13-3 concentration being used. IC_50_ (inhibition of cell viability of 50%) values were calculated from the dose-response curves, an example is given in Figure 3. Statistics: Values are expressed as the mean of three independent experiments ± standard error.

### 3.8. MTT Assay

After trypsinization KB cells were suspended as a 200,000 cells/mL suspension and 50 μL were dropped in each well of 96-well microplates (Costar, Corning, NY, U.S.). Tests were performed once the cells had settled at the bottom of the wells (48 h cultures) by incorporating 50 μL of test solutions. After 72 h of incubation, cell viability was determined using the colorimetric MTT assay according to Denizot and Lang [31]. This test was mainly used for the detection and follow-up of active fractions in the course of purification.

## Figures and Tables

**Table 1:** ^1^H, ^13^C and COSY NMR spectroscopy data for peracetylated F13-3 glycolipids in CDCl_3_.

Position	δ_H_ ppm, mult., *J* in Hz	δ_C_ ppm	cosy correlation
NH	6.35 (d, *J* = 9.0)	-	

1a	3.95 (dd, *J* = 10.0/3.8)	67.2	2, 1b
1b	3.62 (dd, *J* = 10.0/3.8)	67.2	2, 1a
2	4.32 (m)	72.7	1a, 1b, 3, NH
3	4.27 (d, *J* = 4.6)	50.6	4, 2
4	5.34 (m)	128.7	3, 5
5	5.43 (dd, *J* = 6.5/15.0)	124.8	4, 6
6	2.07 (s)	32.3	5, 7
7	2.20 (s)	31.9	6, 8, 19
8	5.83 (m)	136.4	7
9	-	134.2	
10	6.04 (d, *J* = 15.4)	134.4	11
11	5.59 (m)	127.8	10, 12
12	2.10 (m)	32.9	11, 13
19	1.73 (s)	12.5	7, 8
CH_2_	1.27 (m)	22.7–29.7	
CH_3_ acetates	2.02/2.04/2.05/2.08/2.11/2.19 (6s)	20.6–21.0	
C=O acetates		169.3/169.4/169.6/160.7/169.8/169.9	
terminal CH_3_	0.90 (t, *J* = 6.8)	14.1	
1′	4.49 (d, *J* = 7.9)	100.5	2′
2′	4.97 (dd, *J* = 8.0/9.5)	71.2	1′, 3′
3′	5.20 (t, *J* = 9.5)	72.7	2′, 4′
4′	5.10 (t, *J* = 9.7)	68.2	3′, 5′
5′	3.71 (m)	71.9	4′, 6′a, 6′b
6′a	4.24 (d, *J* = 4.5)	61.8	5′, 6′b
6′b	4.16 (dd, *J* = 12.5/2.3)	61.8	5, 6a
1″	-	172.2	
2″	5.36 (m)	73.0	
3″	1.83 (m)	31.8	
4″	1.40 (m)	29.5	
terminal CH_3_	0.83 (m)	14.1	

**Table 2:** IC_50_ measures for F13-3 (μM ± S.E., 24 h of treatment).

IC_50_	KMS-11	HCT-116	GBM
F13-3	15.2 ± 4.0	18 ± 3.9	34.6 ± 5.1

## Abbreviations

COSY: homonuclear correlation spectroscopy
FAME: fatty acid methyl ester
GC-MS: gas chromatography-mass spectrometry
GL: glycolipid(s)
GSL: glycosphingolipid(s)
HMBC: heteronuclear multiple bond coherence
HSQC: heteronuclear single quantum coherence
IC_50_: 50% inhibitory concentration
ESI-MS: electrospray ionization mass spectrometry
TLC: thin layer chromatography
